# Inhibition of TMEM16A by Natural Product Silibinin: Potential Lead Compounds for Treatment of Lung Adenocarcinoma

**DOI:** 10.3389/fphar.2021.643489

**Published:** 2021-04-14

**Authors:** Shuai Guo, Xue Bai, Yufei Liu, Sai Shi, Xuzhao Wang, Yong Zhan, Xianjiang Kang, Yafei Chen, Hailong An

**Affiliations:** ^1^ College of Life Science, Hebei University, Baoding, China; ^2^ Key Laboratory of Molecular Biophysics, Hebei Province, Institute of Biophysics, School of Sciences, Hebei University of Technology, Tianjin, China

**Keywords:** TMEM16A, inhibitor, silibinin, lung adenocarcinoma, molecular target

## Abstract

**Background**: Effective anticancer therapy can be achieved by identifying novel tumor-specific drug targets and screening of new drugs. Recently, TMEM16A has been identified to be overexpressed in lung adenocarcinoma, and inhibitors of TMEM16A showed obvious antitumor efficacy.

**Methods**: YFP fluorescence quenching and whole-cell patch clamp experiments were used to explore the inhibitory effect of silibinin on TMEM16A. Molecular docking and site-directed mutagenesis were performed to confirm the binding sites of silibinin and TMEM16A. MTT assay, wound healing assay, and annexin-V assay were used to detect the effect of silibinin on cancer cell proliferation, migration, and apoptosis. shRNA was transfected into LA795 cells to knock down the expression of endogenous TMEM16A. Tumor xenograft mice combined with Western blot experiments reveal the inhibitory effect and mechanism of silibinin *in vivo*.

**Results**: Silibinin concentration dependently inhibited the whole-cell current of TMEM16A with an IC_50_ of 30.90 ± 2.10 μM. The putative binding sites of silibinin in TMEM16A were K384, R515, and R535. The proliferation and migration of LA795 cells were downregulated by silibinin, and the inhibition effect can be abolished by knockdown of the endogenous TMEM16A. Further, silibinin was injected to tumor xenograft mice which exhibited significant antitumor activity without weight loss. Finally, Western blotting results showed the mechanism of silibinin inhibiting lung adenocarcinoma was through apoptosis and downregulation of cyclin D1.

**Conclusion**: Silibinin is a novel TMEM16A inhibitor, and it can be used as a lead compound for the development of lung adenocarcinoma therapy drugs.

## Introduction

*Cancer* is one of the most serious malignant diseases that threaten the survival of human beings in the world (Allemani et al., 2018). Among all cancer types, lung cancer ranks the first in the incidence and mortality of all cancers (Bray et al., 2018). Currently, surgery is the only effective way to radical cure for lung cancer, but it still needs to be combined with adjuvant chemotherapy after surgery (Aokage et al., 2017). In addition, some lung cancers metastasize early and can be only relied on chemotherapy (Nasim et al., 2019). Therefore, chemotherapy is one of the main methods of treating lung cancer. However, the lung cancer chemotherapy drugs generally have serious side effects (Islam et al., 2019). The emergence of targeted anticancer drugs improved the chemotherapy effect of tumors. Anticancer drugs have good therapeutic effects and few side effects. The disadvantages of targeted anticancer drugs are that they are prone to drug resistance, and it needs to be continuously updated to extend the survival time of patients (Hirsch et al., 2017; Mayekar and Bivona, 2017). Therefore, researchers are constantly exploring new anticancer targets and new drugs.

TMEM16A was a new lung cancer biomarker (Hu et al., 2019). TMEM16A gene was found to be amplified as part of human chromosome 11q13 amplicon in cancers (Qu et al., 2014). This maybe the reason that TMEM16A interacted with many cancers. TMEM16A is closely related to the sustained proliferation of cancer cells (Crottes and Jan 2019). In addition, it also has a relatively important impact on cancer cell proliferation, apoptosis resistance, migration, and invasion (Guo et al., 2017; Wang et al., 2017). Tumor growth can be significantly suppressed by inhibiting the high expression of TMEM16A in the cells (Hu et al., 2019). Research studies showed TMEM16A is hardly expressed in normal lung tissues, but the expression in lung cancer cells increased sharply (Zhang et al., 2020). Lung cancer therapy drugs targeting TMEM16A have little side effects, little resistance, and strong specificity (Guo et al., 2020c). Therefore, the exploring of lung adenocarcinoma targeted drugs which targeting TMEM16A is a new trend in lung adenocarcinoma drug development.

Herbal medicines are a source of drug discovery for lung cancer treatment. A variety of herbal medicine compounds and active ingredients showed satisfactory therapeutic effects to lung cancer. For example, the extract of a polyherbal mixture containing Nigella sativa (seeds), Hemidesmus indicus (roots), and Smilax glabra (rhizomes) showed anti-NSCLC effect (Pathiranage et al., 2020). Six natural products isolated from Carissa carandas showed potent activity against lung cancer (Bano et al., 2021). Silibinin is one of the main effective ingredients of the herbal medicine milk thistle (Di Fabio et al., 2013). Silibinin can protect liver cell membranes, promote the growth of liver cells, enhance the activity of macrophages, promote fat transfer, and reduce liver damage (Singh et al., 2020; Tsaroucha et al., 2020). At present, silibinin is often used to treat hepatitis, cirrhosis, fatty liver, liver poisoning, and other liver diseases clinically (Derakhshandeh-Rishehri et al., 2020; Jia et al., 2020). In addition, silibinin can inhibit the growth and differentiation of several cancer cells, but the molecular mechanism is not yet clear (Sun et al., 2020).

This work newly found that silibinin is an effective TMEM16A inhibitor, and it can inhibit lung cancer growth by inhibiting the endogenic expressed TMEM16A in lung adenocarcinoma. We confirmed the inhibitory effect of silibinin on TMEM16A through fluorescence experiments and patch clamp experiments. Molecular docking and site-directed mutagenesis were combined to find the putative binding sites of silibinin and TMEM16A. The effect of silibinin inhibiting lung adenocarcinoma was verified by *in vitro* and *in vivo* experiments. Finally, we explored the signal transduction mechanism of silibinin anticancer.

## Materials and Methods

### Materials

Silibinin was purchased from Solarbio (CAS No.: 22888-70-6; Beijing, China). RPMI-1640 medium was purchased from Thermo Fisher Scientific (Waltham, United States). Fetal bovine serum (FBS) was purchased from Sijiqing (Hangzhou, China). 3-(4,5-dimethyl-2-thiazolyl)-2,5-diphenyl-2H-tetrazolium bromide (MTT) was purchased from Solarbio (CAS No.: 298-93-1). The TMEM16A antibody (ab53212), MEK1/2 antibody (ab178876), β-catenin antibody (ab223075), and goat antirabbit (IgG) secondary antibody (ab150077) were purchased from Abcam (Cambridge, United Kingdom). The MEK1/2 phospho-antibody (11,205) and ERK1/2 phospho-antibody 12,548) were purchased from Signalway Antibody (Texas, United States). The ERK1/2 antibody (K200062 M) was purchased from Solarbio (Beijing, China). The cyclin D1 antibody (60186-1-Ig) was purchased from Proteintech (Chicago, United States). The N-cadherin antibody (A01577-3), E-cadherin antibody (BM4166), and vimentin antibody (PB9395) were purchased from Boster (Beijing, China). The cleaved-caspase 3 antibody (AF7022) and cleaved-caspase 9 antibody (AF5240) were purchased from Affinity Biosciences (Changzhou, China). Nude mice were purchased from SPF Biotechnology (Beijing, China).

### Cell Culture and Transfection

LA795 cells were maintained under standard cell culture conditions of 5% CO_2_ and 95% humidity. LA795 cells were cultured in RPMI-1640 medium with 10% FBS. The mouse cDNA clone mTMEM16A (accession number NM_178,642.5) was kindly provided by Prof. Young Duk Yang (Seoul National University, Korea). The cells were transfected with lentiviral particles containing shRNA. The following shRNA targeting the mouse TMEM16A gene was used: CCT​GCT​AAA​CAA​CAT​CAT​T (2,399–2,418 nt). The transient transfection of lentiviral particles was performed with X-tremeGENE HP (Roche, Basel, Switzerland).

### Fluorescence Assay

The Premo™ Halide Sensor fluorescence was observed by confocal laser scanning microscopy (CLSM) (Leica SP5, Solms, Germany). YFP-F46L/H148Q/I152 L plasmid was transfected into LA795 cells for 36 h for use. The silibinin solution with a final concentration of 100 μM was added to the 24-well plate to incubate the cells for 30 min. Then, the cells were washed three times with D-PBS solution, and 500 μL D-PBS was left in the well. The 24-well plate was placed to the objective table and the fluorescence was tested with a 488 nm excitation light and 520 ± 15 nm emitted light. 500 μL D-PBS which contained 150 mM I^−^ was added to each well after the fluorescence intensity curve stabilized. A live photo was taken every 1.5 s with xy-t mode of CLSM (200 photos were taken continuously).

D-PBS buffer solution: 2.67 mM KCl, 1.47 mM KH_2_PO_4_, 138 mM NaCl, 8.1 mM Na_2_HPO_4_, and adjusted to pH 7.4 using NaOH.

### Electrophysiology

All patch clamp experiments were performed at 22–25°C. Patch pipettes were pulled from borosilicate glass capillaries (Sutter Instrument, Novato, CA) using a P-97 puller (Sutter) with a pipette resistance of 3–5 MΩ when immersed in the bath solution. Data recordings were performed with an EPC10 amplifier controlled by Pulse software with a Digi LIH1600 interface (HEKA Elektronik, Lambrecht, Germany). The data were recorded after seal resistance of the patch clamp reached 1 GΩ or higher. The data were low-pass filtered at 2.9 kHz and sampled at 10 kHz. The stimulation protocol consisted of voltage steps of 1,150 ms durations from a holding potential of 0 mV for 100 ms; the membrane voltage was clamped in steps of 20 mV from -80 to +80 mV for 750 ms, then back down to −80 mV for 300 ms.

Pipette solution: 130 mM CsCl, 10 mM EGTA, 1 mM MgATP, 1 mM MgCl_2_.6H_2_O, 10 mM HEPES, and adjusted to pH 7.4 with CsOH. Bath solution: 150 mM NaCl, 1 mM MgCl_2_.6H_2_O, 10 mM HEPES, 10 mM glucose, 10 mM mannitol, and adjusted to pH 7.4 with NaOH. The Ca^2+^ concentrations in the pipette solution were calculated using the CaEGTA Calculator v1.2: http://www.stanford.edu/∼cpatton/CaEGTA-NIST.htm. The osmotic pressure of the pipette solution was 290–300 mOsm/L and bath solution was 300–310 mOsm/L (measured by OM815 osmometer, LöserMesstechnik, Germany).

### Modeling and Molecular Docking

The structure of the mTMEM16A was constructed using a calcium-bound mTMEM16A chloride channel (PDB ID: 5oyb) as described previously (Guo et al., 2019b; Guo et al., 2020a). The SWISS-MODEL server was used to complement the missing structure of 5oyb. A two-step molecular docking strategy was used in this work which combined global random docking with local docking. First, 100 times of global random docking were performed without any restrictions. Second, a 35 Å × 35 Å×35 Å domain was selected for local docking which is centered on the area where silibinin was mostly distributed in the first step. Molecular docking of silibinin to mTMEM16A was performed with an AutoDock 4.2 program using the implemented empirical free energy function and the Lamarckian genetic algorithm. The initial coordinates of the ligand were mapped using ChemBioDraw Ultra 12.0. Visualization and analysis of complexes was performed using VMD 1.9 and PyMOL 0.99.

### Site-Directed Mutagenesis

The site-directed mutagenesis primers were designed by Agilent Primer Design Website (https://www.agilent.com/store/primerDesignProgram.jsp): K384 A Primer, 5′-gtg​tcc​tct​gtg​tga​cgc​gac​ctg​cag​cta​ctg​g-3’. R515 A Primer, 5′-aat​cgt​cct​cgg​agt​tat​cat​cta​tgc​aat​ctc​cac​agc​tg-3’. R535 A Primer, 5′-gtg​cgg​tcc​aac​atc​gcg​gtt​aca​gtc​acg​gc-3’. Primer synthesis and sequencing were completed by Sangon (Shanghai, China). Transgen Fast Mutagenesis System Kit (FM111–02; Beijing, China) was used to conduct site-directed mutagenesis with a 50 μL PCR system.

### Wound Healing Assay

LA795 cells were cultured to 90% confluence in 6-well plate. The cells were scraped with a sterile 10 μL tip and washed twice with D-PBS. Then, culture with RPMI-1640 medium containing 1% FBS followed by treatment with 0–300 μM silibinin or DMSO (as control). The plates were photographed at 0, 24, 48, and 72 h using a Nikon microscope (TE2000-S, Tokyo, Japan) at ×100 magnification. ImageJ software (National Institutes of Health, Bethesda, United States) was used to calculate the wound healing area. The relative scratch area was determined by the experimental group to that in the control group.

### MTT Assay

4,000–7,000 LA795 cells were seeded to each well of the 96-well plate and cultured for 24 h. DMSO (as control) or silibinin (0–300 μM) was added to the cells for culture. Then, 5 mg/ml MTT working solution was added to the cells to incubate for 4 h. The supernatant of the medium was discarded, and 150 μL DMSO was added to each well. The 96-well plate was placed on a shaker at 30 rpm for 10 min to fully dissolve the crystals. The absorbance of cells at 490 nm was recorded using a molecular device (SpectraMAX i3; Sunnyvale, United States). The percentage of cell viability was calculated by dividing the absorbance of the silibinin-treated group by the control group.

### Annexin-V Assay

Cell apoptosis was detected with the annexin V-FITC Apoptosis Detection Kit (CA1020, Solarbio, Beijing, China). LA795 cells were seeded in 6-well culture plates for 24 h. Then, the cells were incubated with silibinin (200 μM) for 24 h. Cells were trypsinized and suspended in 500 µL of binding buffer containing 5 µL annexin V-FITC and 5 µL propidium iodide (PI). Cells were analyzed by using a CytoFLEX flow cytometer (Beckman Coulter, California, United States).

### Tumor Xenografts in Mice

5 × 10^6^ LA795 cells were inoculated to nude mice (6–8 week old) in the right forelimb. Tumor volumes were measured every 3 days using a vernier caliper, and volumes of tumor were calculated using a standard formula (length × width^2^/2). The mice were divided into four groups when the tumor volume reached 150 mm^3^: 1) control group, 2) cisplatin group (7.5 mg/kg/kg body weight (BW)/3 days/tail vein), 3) silibinin (50 mg/kg body weight (BW)/3 days/tail vein), and 4) silibinin (100 mg/kg body weight (BW)/3 days/tail vein). Drug administration to mice via tail vein injection. Silibinin was dissolved in DMSO to prepare a 1 M solution. Then, the silibinin solution was diluted with normal saline to the concentration required for the experiments. The mice were sacrificed after injecting 10 times.

### Western Blotting

Different groups of LA795 cells were washed three times with ice D-PBS solution and then lyzed on ice for 30 min. The proteins were separated on 10% sodium dodecyl sulfate–polyacrylamide gels and electroblotted onto a nitrocellulose membrane in 25 mM Tris base and 190 mM glycine at 100 V for 3 h at 4°C. Blots were incubated in a 1:1,000 dilutions of the corresponding monoclonal antibodies against TMEM16A, β-caterin, E-cadherin, N-cadherin, vimentin, MEK1/2, *p*-MEK1/2, ERK1/2, *p*-ERK1/2, cyclin D1, cleaved-caspase 3, cleaved-caspase 9, and β-actin for 12 h at 4°C. The membranes were then probed with the immunoreactivity by adding secondary antibody diluted to 1:5,000 detecting it with chemiluminescent HRP detection kit.

### Data Analysis

Origin 8.0 and GraphPad Prism 8.0.1 were used for graphic creation and statistical data analysis. All data were presented as mean values ±SE. Statistical significance between two groups was determined using independent *t*-test. Asterisks indicate significant differences (*p < 0.05, **p < 0.01). The capacitive transients of some traces in the figures were trimmed for clarity.

## Results

### Silibinin Was a Novel TMEM16A Inhibitor

**FIGURE 1:**
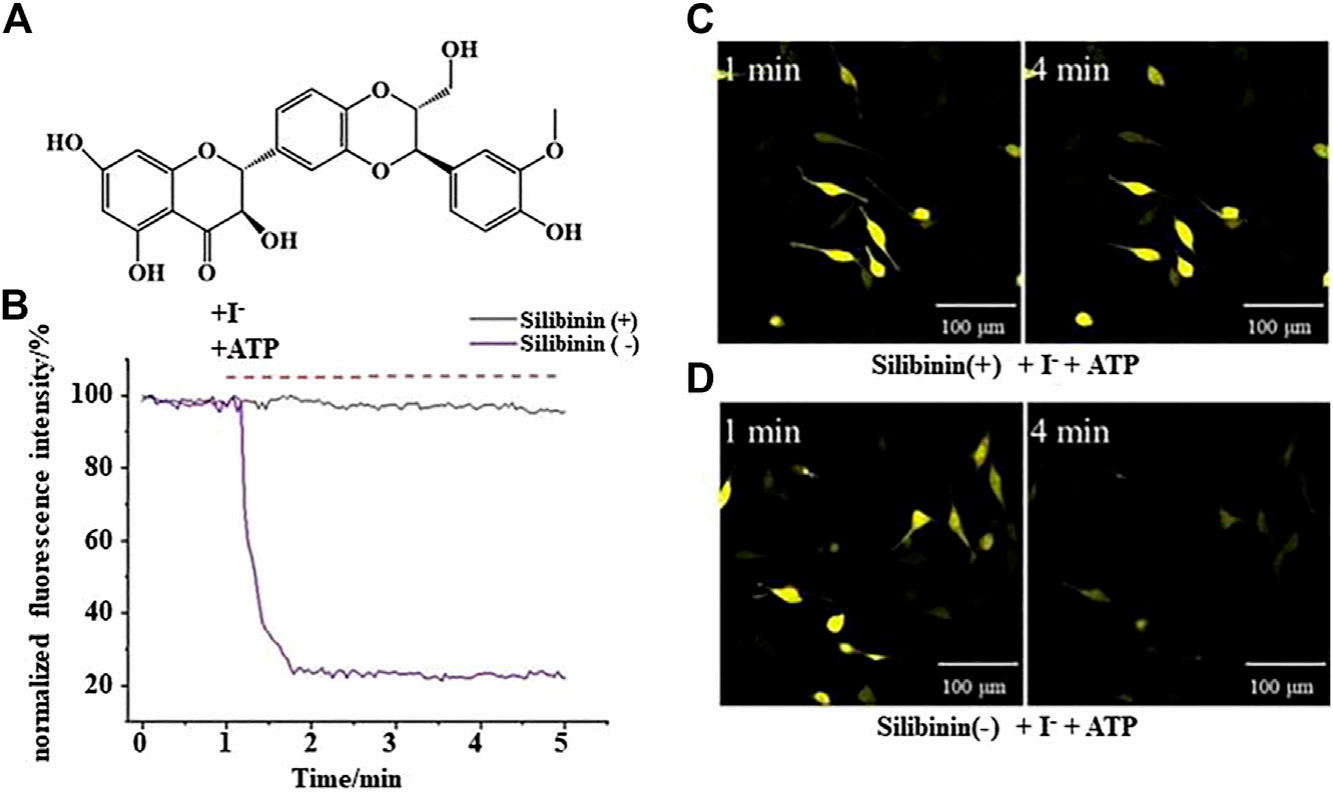
Silibinin inhibited TMEM16A to prevent YFP fluorescence quenching **(A)**. Molecular structure of silibinin. **(B)**. YFP fluorescence quenching curve caused by ATP in the presence (or absence) of silibinin (*n* = 3). **(C) and (D)**. Representative real-time fluorescence images of LA795 cells incubated with (or without) silibinin (*n* = 3).

YFP fluorescence quenching experiments were conducted to screen new TMEM16A inhibitors. The results showed that silibinin was a novel TMEM16A inhibitor. Figure 1A shows the molecular structure of silibinin. Figure 1B showed that the quenching of YFP fluorescence caused by ATP in LA795 cells can be abolished by silibinin. Real-time fluorescence images showed that the fluorescence intensity of the silibinin incubation group unchanged, while the fluorescence intensity of the control group almost completely quenched after 4 min of adding ATP (Figures 1C,D). The above results indicated that silibinin was an effective TMEM16A inhibitor which can prevent I^−^ flow into the cell from the bath solution by closing the TMEM16A on LA795 cell membrane.

### Silibinin Concentration dependently Inhibited TMEM16 A Whole-Cell Currents

**FIGURE 2:**
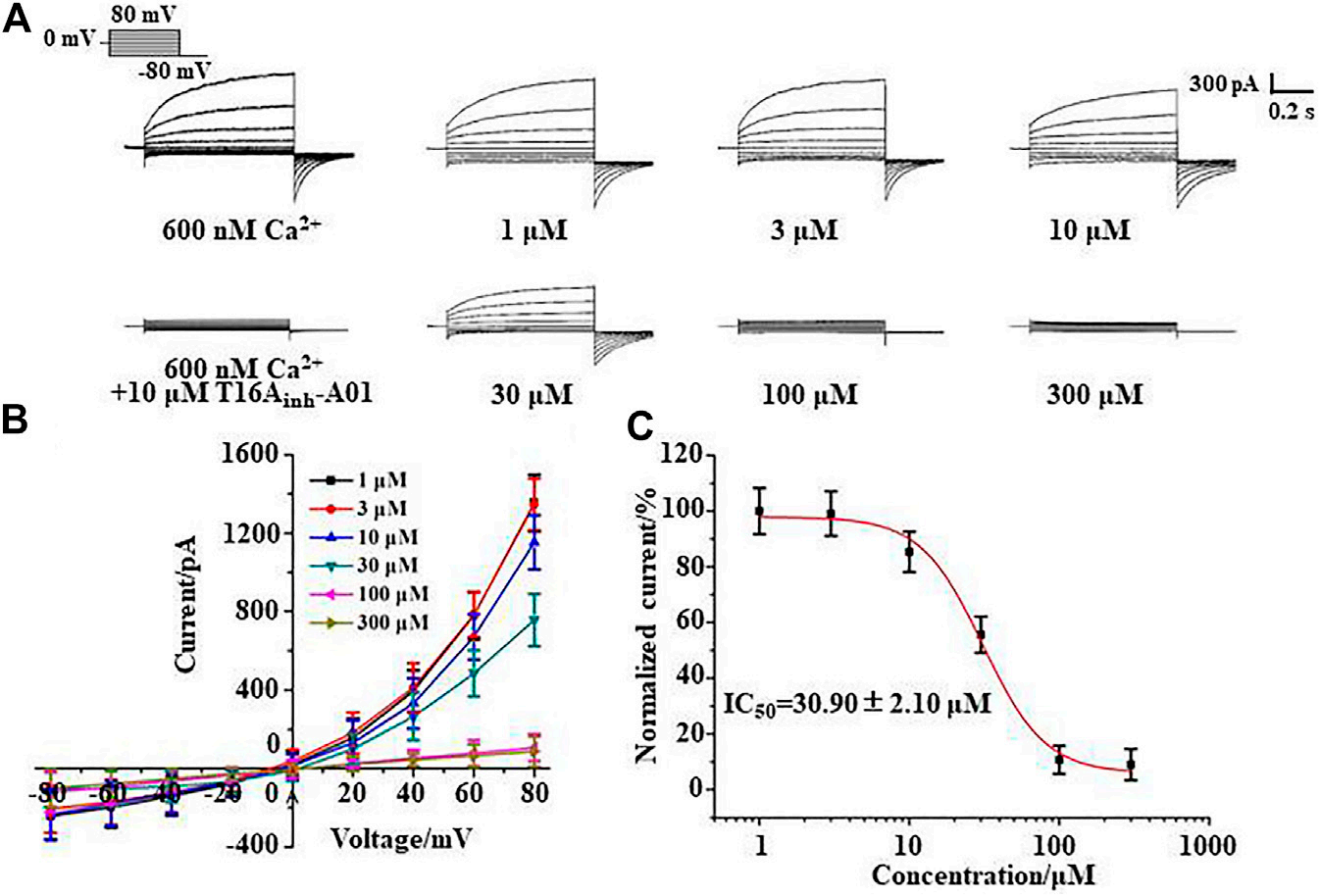
Silibinin inhibited TMEM16A whole-cell currents in a concentration-dependent manner. **(A)**. Typical currents of TMEM16A activated by 600 nM Ca^2+^ and inhibited by T16A_inh_-A01, or different concentrations of silibinin (*n* = 6). **(B)**. I-V curve of the TMEM16A currents inhibited with different concentrations of silibinin (*n* = 6) **(C)**. Dose-response curve of TMEM16A currents inhibited by silibinin in LA795 cells (*n* = 6).

Whole-cell patch clamp experiments were performed to study the inhibitory effect of silibinin on TMEM16A. The pipette solution containing 600 nM Ca^2+^ can activate the TMEM16A in LA795 cells to generate currents of about 1,400 pA. This TMEM16A current can be almost completely inhibited by TMEM16A-specific inhibitor T16A_inh_-A01 (Figure 2A). The TMEM16A whole-cell currents significantly reduced when silibinin was added into the bath solution. However, the currents still showed activate slowly with time and outward rectification with a deactivating tail current on repolarization characteristics. Subsequently, we performed a statistical analysis of the current–voltage relationship (I-V curve) of silibinin inhibiting TMEM16A currents under different clamping voltages (Figure 2B). I-V curve results showed that silibinin inhibited TMEM16A whole-cell currents in a concentration-dependent manner. Next, different concentrations of silibinin inhibited the TMEM16A current at +80 mV were extracted, and the data were fitted by Hill equation to obtaine the dose-effect curve of silibinin inhibiting TMEM16A currents (Figure 2C). The above results indicated that silibinin inhibited TMEM16A whole-cell currents in a concentration-dependent manner with an IC_50_ of 30.90 ± 2.10 μM.

### The Putative Binding Sites of Silibinin and TMEM16A

**FIGURE 3:**
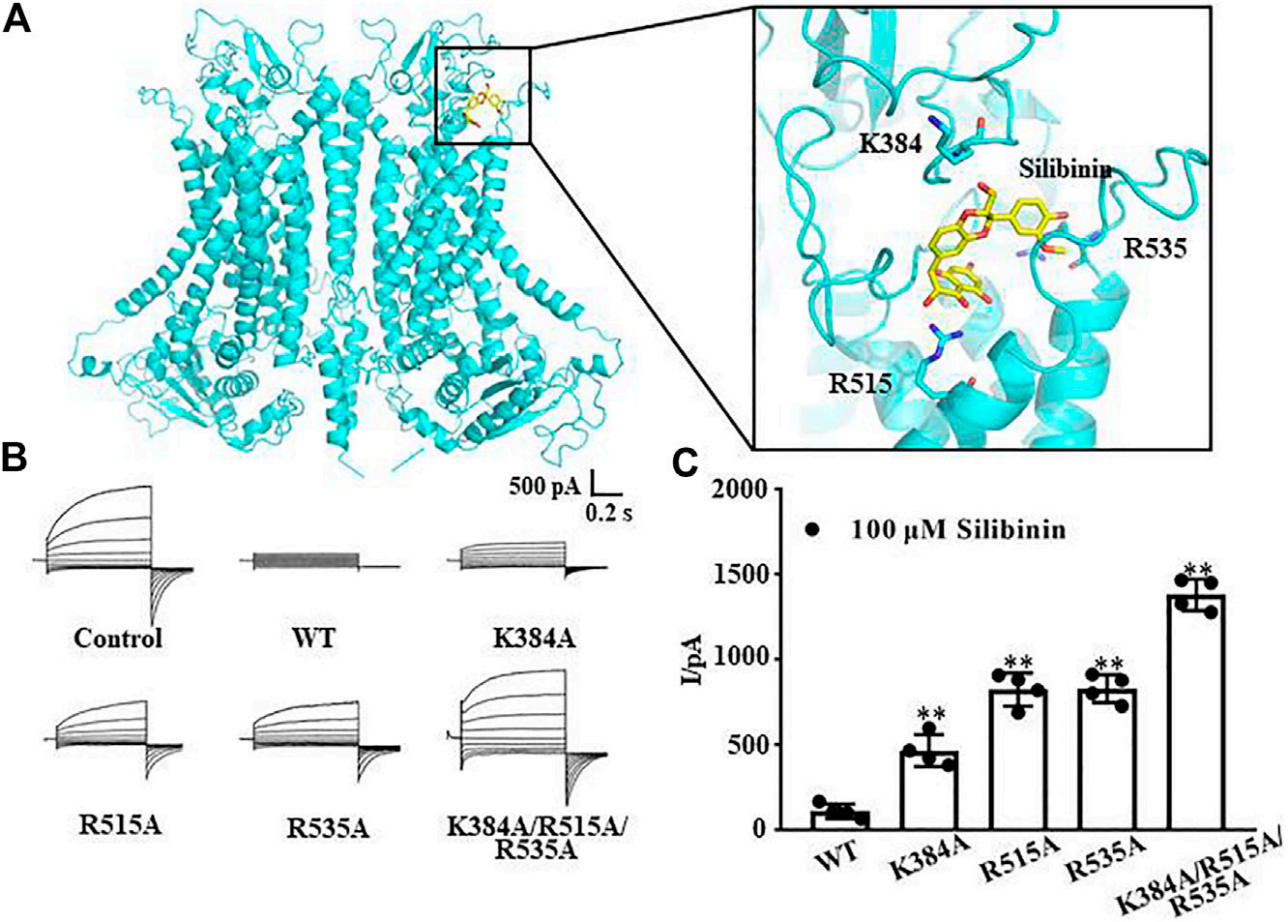
Putative binding sites of silibinin combined to TMEM16A. **(A)**. Binding region and putative binding sites of silibinin combined with TMEM16A predicted by molecular docking. **(B)**. Typical currents of silibinin inhibited wild-type and mutants TMEM16A currents activated by 600 nM Ca^2+^ (*n* = 4). **(C)**. Statistical results of currents at +80 mV in (B) (*n* = 4).

The molecular docking results showed that the putative binding sites of silibinin and TMEM16A were mainly distributed in the entrance of the TMEM16A protein near the pore outside. Further analysis showed that the amino acid residues K384, R515, and R535 have the strongest interaction with silibinin (Figure 3A). Therefore, we mutated the positively charged lysine and arginine to uncharged alanine and performed whole-cell patch clamp experiments again. The results showed that 100 μM silibinin, which can almost completely inhibit the wild-type TMEM16A currents, can only inhibit part of the currents of the single mutants; moreover, it can hardly inhibit the three mutant currents (Figure 3B). Through the above experiments, we confirmed that K384, R515, and R535 were the binding sites of silibinin and TMEM16A which laid a foundation for studying the pharmacological properties of silibinin.

### Silibinin Significantly Inhibited the Proliferation, Migration, and Promoted the Apoptosis of Lung Cancer Cells

**FIGURE 4:**
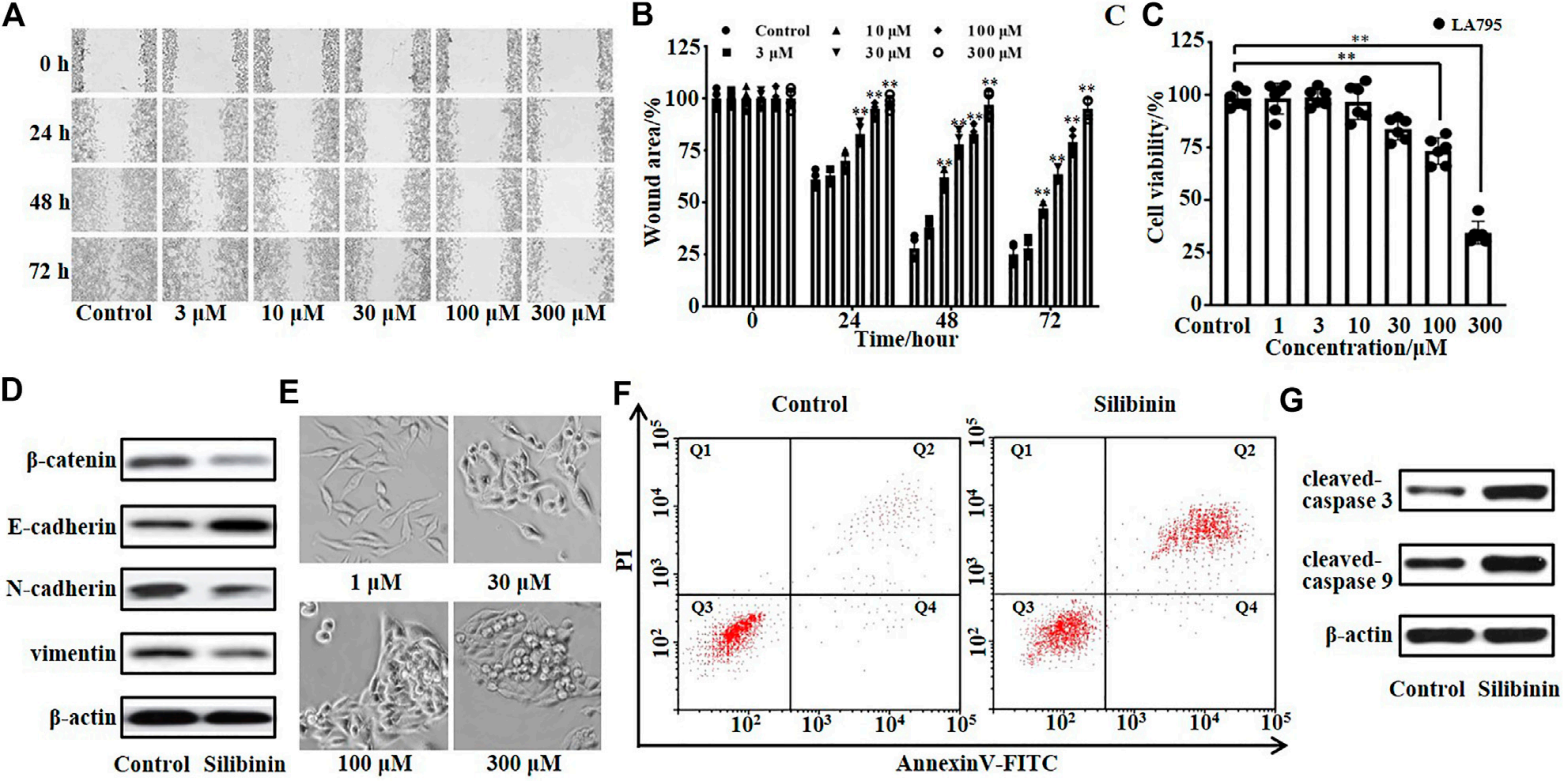
Silibinin inhibited the growth and promoted the apoptosis of LA795 cells. **(A)**. Migration of LA795 cells in the presence of 0, 3, 10, 30, 100, and 300 μM silibinin assessed by wound healing assay (*n* = 4). **(B)**. Statistical results of the percentage of wound area in **(A)** (*n* = 4) **(C)**. Statistical results of LA795 cells viability incubated with different concentrations of silibinin (*n* = 6). **(D)**. Expression of *β*-catenin, E-cadherin, N-cadherin, and vimentin of LA795 cells after treated with 200 μM silibinin for 24 h (*n* = 3). **(E)**. Representative real-time images of LA795 cells incubated with different concentrations of silibinin (*n* = 6). **(F)**. Representative images of apoptotic cells after 200 μM silibinin treatment for 24 h detected by annexin V apoptosis assay (*n* = 3). **(G)**. Expression of cleaved-caspase 3 and cleaved-caspase 9 of LA795 cells after 200 μM silibinin treatment for 24 h (*n* = 3).

TMEM16A was endogenously highly expressed in lung adenocarcinoma LA795 cells, and TMEM16A was closely related to the growth of cancer cells. Therefore, we tested the inhibitory effect of silibinin on the growth of LA795 cells by MTT, wound healing and annexin-V assays. Real-time images of wound healing assay showed that silibinin can significantly inhibit the migration of LA795 cells (Figure 4A). Statistical analysis of the abovementioned results showed the inhibitory effect of silibinin to LA795 cell migration was both time- and concentration-dependent (Figure 4B). MTT experiments showed that silibinin inhibited the proliferation of LA795 cells in a concentration-dependent manner (Figure 4C). In addition, we performed Western blot experiments with another two lung cancer cell lines (A549 and NCI-H1299). The results showed TMEM16A was highly expressed in the cells (Supplementary Figure S1A). The results of wound healing and MTT experiments showed that silibinin significantly inhibited the migration and proliferation of A549 and NCI-H1299 cells (Supplementary Figures S1B–S1G).

Then, we detected the key proteins related to cell invasion by Western blot. The results showed β-catenin, N-cadherin, and vimentin were reduced, while E-cadherin was increased after silibinin incubation (Figure 4D). The real-time images showed the high concentration of silibinin was toxic to LA795 cells which caused the cell morphology shrank to death (Figure 4E). Therefore, we tested the effects of silibinin on the cell apoptosis through annexin V-FITC/PI staining assay (Figure 4F). 200 μM silibinin promoted the apoptosis of LA795 cells with 57.2 ± 2.6%. At the same time, the apoptotic protein cleaved-caspase 3 and cleaved-caspase 9 significantly increased after silibinin treatment of cells (Figure 4G).

### TMEM16A Was the Drug Target for Silibinin Inhibiting the Growth of LA795 Cells

**FIGURE 5:**
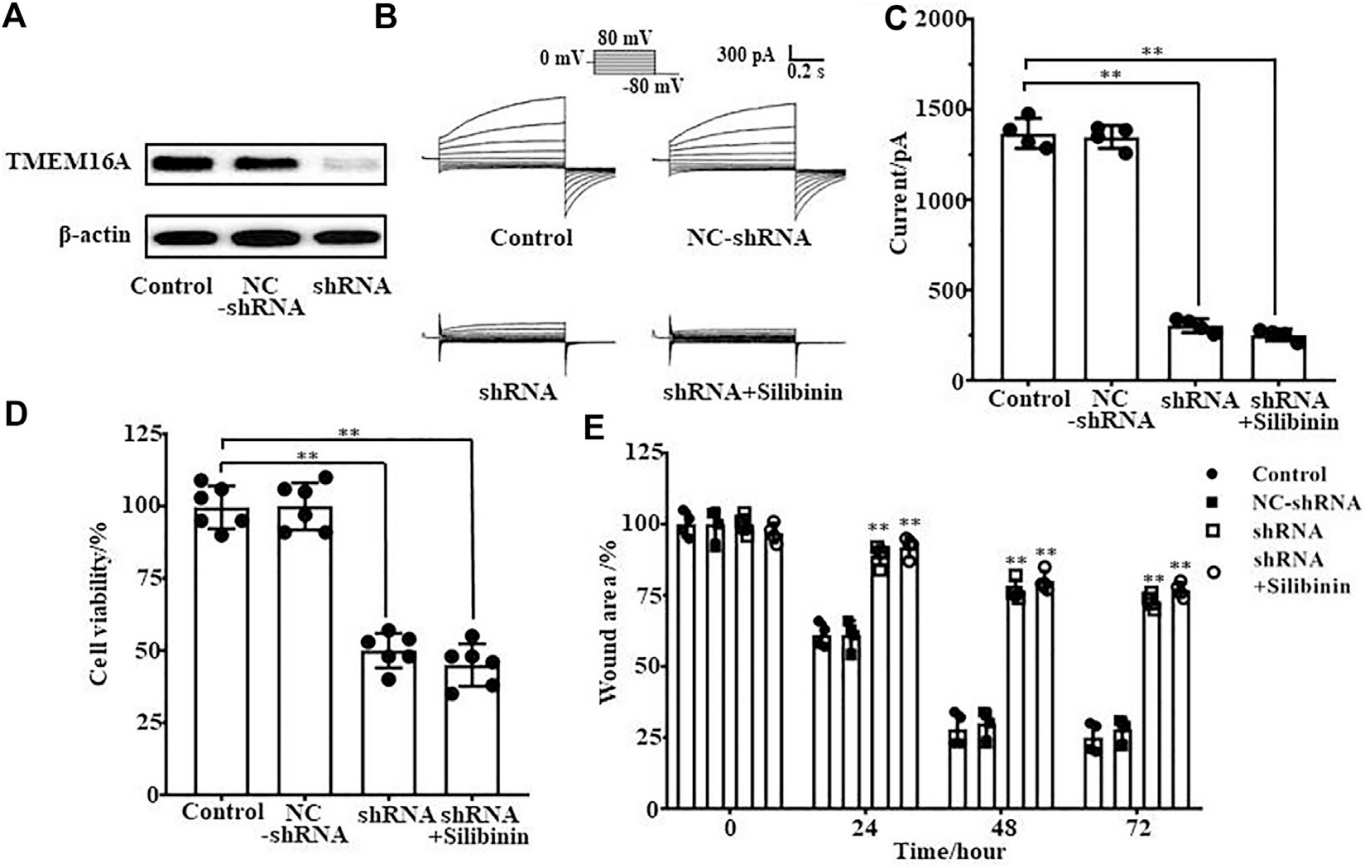
Silibinin inhibited the proliferation and migration of LA795 cells by inhibiting TMEM16A. **(A)**. Expression of TMEM16A in LA795 cells after transfected NC-shRNA or shRNA for 24 h (*n* = 3). **(B)**. Typical currents of TMEM16A negative control shRNA, shRNA and shRNA added with 100 μM silibinin. All the currents were activated by 600 nM Ca^2+^ in LA795 cells (*n* = 4). **(C)**. Statistical results of currents at +80 mV in (B) (*n* = 4). **(D)**. Statistics results of cell viability after transfection with TMEM16A shRNA and addition of silibinin (*n* = 6). **(E)**. Statistics results of cell wound area after transfection with TMEM16A shRNA and addition of silibinin (*n* = 4).

As silibinin can inhibit both TMEM16A currents and LA795 cell growth, we hypothesized that TMEM16A was the drug target for silibinin inhibiting the growth of LA795 cells. TMEM16A shRNA was used to knockdown the endogenous TMEM16A in LA795 cells to verify the hypothesis. Western blot results showed that the expression of TMEM16A in cells transfected with shRNA was significantly reduced (Figure 5A). Whole-cell patch clamp experiments showed that shRNA significantly reduced the endogenous currents of TMEM16A in LA795 cells, and the inhibition of silibinin hardly decreased when shRNA expressed (Figures 5B, C). MTT and wound healing experiments showed the proliferation and migration of LA795 cells with TMEM16A shRNA expression were significantly reduced which proved that TMEM16A plays key role in the proliferation and migration of LA795 cells. The addition of silibinin to LA795 cells with TMEM16A shRNA expression not further reduced their proliferation and migration, which indicated that TMEM16A was the key drug target for silibinin inhibiting the growth of LA795 (Figures 5D,E).

### Silibinin Inhibited the Growth of Lung Adenocarcinoma *in vivo*

**FIGURE 6:**
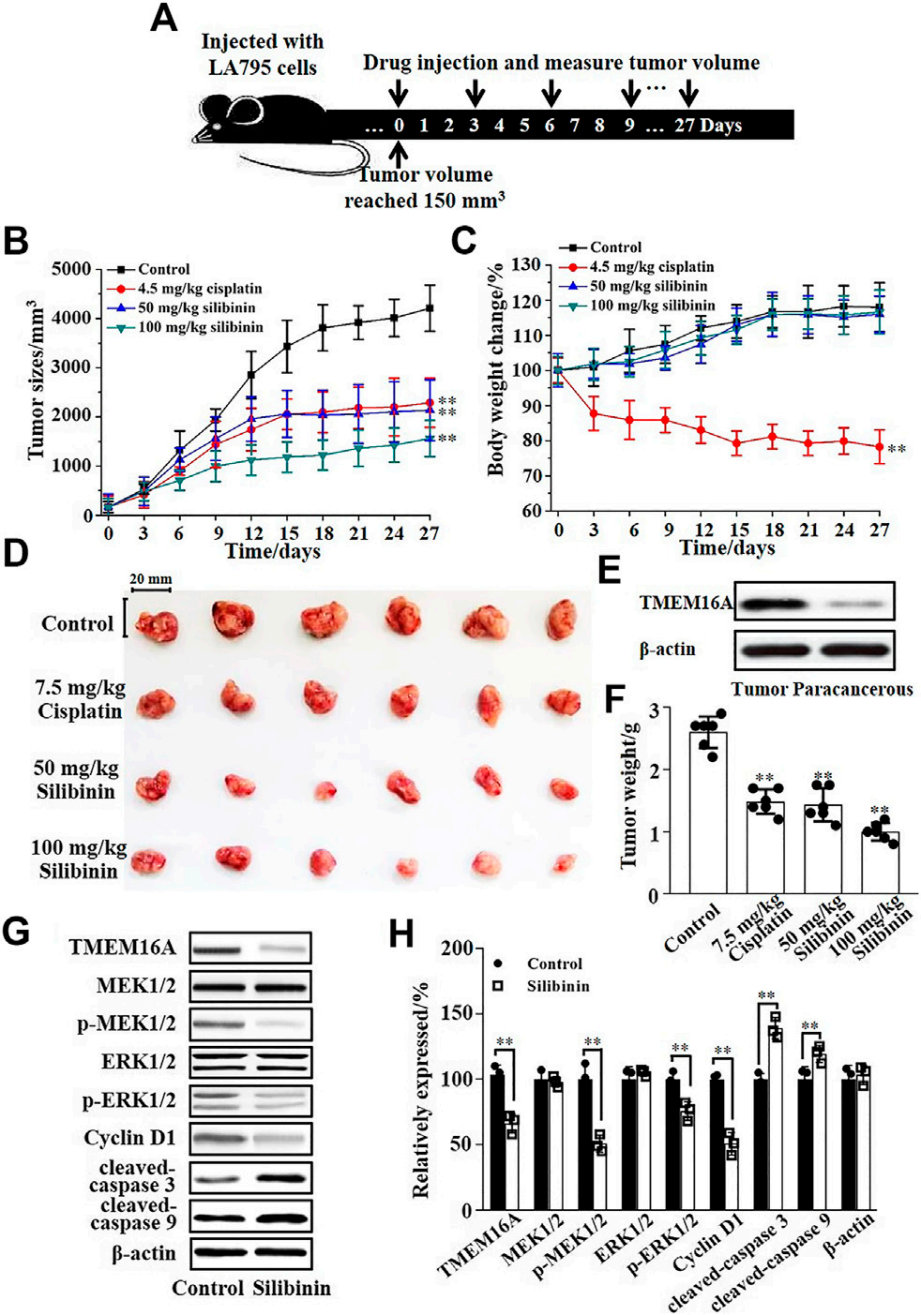
Silibinin inhibited the growth of lung adenocarcinoma in mice and its molecular mechanism. **(A)**. The schematic diagram of the experimental protocol. The xenograft model mice were inoculated with 5 × 10^6^ LA795 cells in the right forelimb, tumor measurements were obtained, and the drugs were subcutaneously injected every 3 days after the tumor volume reached 150 mm^3^. **(B)**. Tumor volume growth curve in different groups (*n* = 6). **(C)**. Body weight growth curve in different groups (*n* = 6). **(D)**. Stripped images of the tumor entity after 10 administrations (*n* = 6). **(E)**. Expression of TMEM16A in tumor tissue and paracancerous tissue (*n* = 3). **(F)**. Statistical results of the stripped tumor weight in **(D)** (*n* = 6). **(G)**. Expression of TMEM16A, MEK1/2, *p*-MEK1/2, ERK1/2, *p*-ERK1/2, cyclin D1, cleaved-caspase 3, and cleaved-caspase 9 in tumor tissue of control and silibinin groups (n = 3). **(H)**. Statistical results of **(G)** (*n* = 3).

The inhibitory effect of silibinin on tumor growth *in vivo* was tested by injecting silibinin into the tail vein of tumor model mice. Figure 6A shows the protocol of the experimental process. Statistical analysis showed that 50 mg/kg silibinin can achieve the same tumor suppressor effect as 7.5 mg/kg cisplatin. When the concentration of silibinin reached 100 mg/kg, the anticancer effect far exceeded that of 7.5 mg/kg cisplatin (Figure 6B). However, the mice became intolerant and lethal when the dose of cisplatin increased to double. The body weight of mice during the administration process was monitored, and the curve showed that 50 mg/kg and 100 mg/kg silibinin had little side effects on mice, while cisplatin significantly reduced the body weight of mice (Figure 6C). The mice were sacrificed after injecting 10 times, and the tumors were dissected (Figure 6D). The Western blot results showed that the expression of TMEM16A in tumor tissues was significantly higher than that in paracancerous tissues (Figure 6E). The tumor weighing statistics showed that the inhibition rates of 50 mg/kg and 100 mg/kg on mice were 46% and 62%, respectively. The inhibition rates were better than that of cisplatin (Figure 6F). Based on the above results, we proposed that silibinin was an effective and safe treatment drug for lung adenocarcinoma.

Western blotting experiments of the tumors were performed to explore the mechanism of silibinin inhibiting the growth of mice lung adenocarcinoma. Western blotting results are shown in Figure 6G; silibinin inhibited the expression of TMEM16A in lung adenocarcinoma, thereby inhibited the phosphorylation of MEK1/2 and ERK1/2, and further reduced the expression of cyclin D1 which led to cancer growth retarded. In addition, silibinin promoted the apoptosis of lung adenocarcinoma cells. The expression of cleaved-caspase 3 and cleaved-caspase 9 increased significantly after silibinin treatment (Figure 6G). The statistical analysis of the protein expression changes is shown in Figure 6H. The above results indicated that the important way that silibinin inhibited the growth of xenografts partially via inhibition of proliferation, downregulation of cyclin D1, and apoptosis.

## Discussion

In this work, we found that silibinin, an active ingredient of Chinese medicine milk thistle, was a novel TMEM16A inhibitor. We confirmed the inhibitory effect of silibinin on TMEM16A by fluorescence experiments and patch clamp experiments. The binding sites of silibinin and TMEM16A were studied by molecular docking and site-directed mutagenesis experiments. Subsequently, it was verified that TMEM16A was the drug target that silibinin inhibited the growth of lung adenocarcinoma *in vitro* and *in vivo*, and the signaling pathway of silibinin inhibited the growth of lung adenocarcinoma was explored.

Silibinin was a newly discovered inhibitor of TMEM16A. This work confirmed that the binding sites of silibinin and TMEM16A for the first time. The three amino acid residues K384, R515, and R535 were located at the entrance of the TMEM16A protein near the pore outside in the cell membrane. The binding sites of a variety of TMEM16A inhibitors were compared, and we found that there are many inhibitors binding with TMEM16A in this region, including CaCC_inh_-A01, arctigenin, and matrine (Guo et al., 2019a; Guo et al., 2020b; Shi et al., 2020). Moreover, the binding sites of many inhibitors to TMEM16A included two same amino acid residues R515 and R535. Therefore, we thought that there may be an inhibitor binding pocket in this region of TMEM16A. Among them, the most critical amino acids for drug interaction included the two polar arginines, R515 and R535. Drugs combined with amino acids in this region of TMEM16A by hydrogen bonds, hydrophobic interactions, and other weak interactions to form a stable conformation, thereby blocking the pores of TMEM16A and preventing chloride ions transported across the membrane. This hypothesis has important guiding significance for the exploration of TMEM16A inhibitors and the development of drugs targeting TMEM16A. It needs more research studies to verify in the future.

TMEM16A was the receptor for silibinin on the membrane of lung adenocarcinoma cells. Although some studies showed that silibinin was a broad-spectrum cancer treatment drug (Bosch-Barrera et al., 2017), most of these studies were focused on the influence of silibinin on signal pathways in apoptosis and cell cycle, but there was lack of discovery of receptors of silibinin (Jahanafrooz et al., 2018). TMEM16A is directly related to multiple signal transduction pathways such as cancer cell proliferation, migration, and invasion in cancer cells (Jia et al., 2015). For example, TMEM16A and EGFR can forming a functional complex to regulate cancer cell proliferation (Bill et al., 2015). Therefore, confirming TMEM16A was the receptor of silibinin on the cell membrane filled the gap between silibinin and the signaling pathway.

Silibinin was a safe lead compound for the lung adenocarcinoma therapy. The chemotherapy drugs of lung cancer treatment in clinically included broad-spectrum anticancer drugs such as cisplatin, carboplatin, and paclitaxel (Teng et al., 2018; Kenmotsu et al., 2019), and targeted anticancer drugs such as gefitinib, afatinib, and osimertinib (Mok et al., 2018; Soria et al., 2018). However, both broad-spectrum anticancer drugs and targeted anticancer drugs have many side effects. For example, broad-spectrum anticancer drugs caused clinically impaired liver and kidney function, neurotoxicity, gastrointestinal dysfunction, myelosuppression, and other side effects (Bisch et al., 2018; Ghosh, 2019). The commonest side effects of targeted anticancer drugs were diarrhea, rash, dry skin, paronychia, nausea, anorexia, mouth ulcers, and so on (Kobayashi et al., 2016). The side effects of targeted drugs were lesser than broad-spectrum drugs, but targeted drugs are prone to drug resistance and need to constantly replace drugs (Gu et al., 2016). Long-term chemotherapy was greatly harmful to the patient's physiology and psychology. Currently, silibinin was a drug used to treat hepatitis clinically without side effects. (Abenavoli et al., 2018). In addition, many studies reported that silibinin had liver protective function; it can resist oxidation and promote the proliferation of liver cells (Liu et al., 2019; You et al., 2020). Furthermore, our *in vivo* experiments showed that silibinin was safe to mice growth. Low-dose silibinin can achieve the same therapeutic effect as cisplatin, but the side effects were much less than cisplatin, and the therapeutic effect of high-dose silibinin was better than cisplatin with also little side effects.

In addition, the duration of action of silibinin was long, which was one of its important advantages as an anticancer lead compound. Cell proliferation and migration experiments showed that the inhibition efficiency of silibinin to cells lasted for 72 h, and its inhibitory effect was increased over time without attenuation. According to the results, we designed the protocol for *in vivo* experiments to inject silibinin into mice every 3 days. The results showed that this long interval administration achieved a good effect. Therefore, we believed that silibinin was metabolized slowly in the mice body and it had a longer duration of action. Long duration means that the patients need longer to take the medication which can greatly relieve the pain of treatment.

The anticancer mechanism of silibinin was varied, including cell proliferation, invasion, and apoptosis. This work showed that silibinin was different from other anticancer drugs which were only cytotoxicity to cell death (Swanepoel et al., 2019). It also prevented the protein phosphorylation of MEK1/2 and ERK1/2 through inhibiting TMEM16A expression and finally arrested the cancer cells in G0/G1. Silibinin regulated β-catenin, E-cadherin, N-cadherin, and vimentin proteins in lung adenocarcinoma cells inhibited cancer cell invasion. In addition, it promoted the apoptosis of lung adenocarcinoma cells. Silibinin targeted the new lung adenocarcinoma drug target TMEM16A which was highly expressed in lung adenocarcinoma but not in normal lung cells. Therefore, silibinin can be used as a new targeted drug with clear binding sites, strong targeting, and high development value.

In summary, silibinin is a novel TMEM16A inhibitor. Meanwhile, it is a safe, efficient, and clear molecular mechanism lead compound for lung adenocarcinoma therapy. The study of silibinin provides new ideas for the development of targeted drugs for lung adenocarcinoma.

## Data Availability

The datasets presented in this study can be found in online repositories. The names of the repository/repositories and accession number(s) can be found in the article/Supplementary Material.

## Abbreviation

CLSM, confocal laser scanning microscopy; DMEM, dimethyl sulfoxide; FBS, fetal bovine serum; LGA, lamarckian genetic algorithm; MTT, 3-(4,5-dimethyl-2-thiazolyl)-2,5-diphenyl-2H-tetrazolium bromide; NSCLC, non-small cell lung cancer; RMS, root mean square
