# Supplementary figures and images for: Inhibition of TMEM16A by Natural Product Silibinin: Potential Lead Compounds for Treatment of Lung Adenocarcinoma
Source: Front Pharmacol. 2021 Apr 14;12:643489. doi: 10.3389/fphar.2021.643489 (PMC8079988; doi:10.3389/fphar.2021.643489)

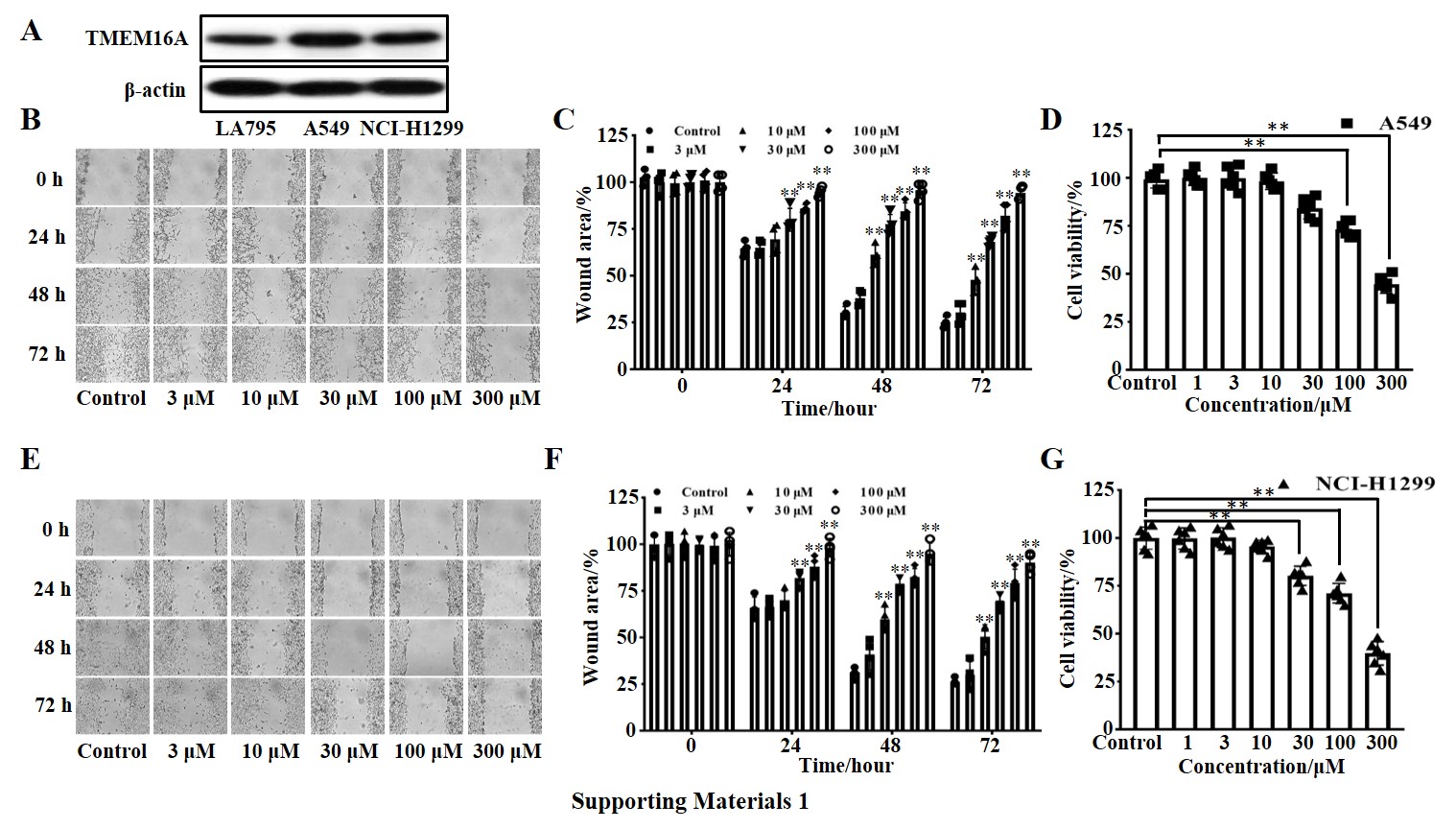

Supplement: Supplementary file 1 [file image1.jpeg]
